# Evidence for subcritical rupture of injection-induced earthquakes

**DOI:** 10.1038/s41598-020-60928-0

**Published:** 2020-03-04

**Authors:** Beata Orlecka-Sikora, Szymon Cielesta

**Affiliations:** Institute of Geophysics, Polish Academy of Sciences, Ks. Janusza 64 Str., 01-452 Warsaw, Poland

**Keywords:** Environmental impact, Seismology

## Abstract

Seismicity induced by geo-engineering operations may be hazardous for people, infrastructure and the environment. The crucial information for assessing induced seismic hazards and related risks is knowledge of the time-dependent strength of rocks and the deformation due to fluid injection. Our studies of seismic and injection data from a geothermal field indicate that pressurized injections lead to rock fracturing at stress levels below the rock toughness, i.e., subcritical fracture growth. We provide a relation between the rate of this subcritical fracture growth and the injection rate. Based on this relation, we estimate the maximum subcritical magnitude. We hypothesize that subcritical fracture growth may be controlled by the amount of stress asymmetry, i.e., the relative values of the principal stresses. We discuss the conditions under which the subcritical fracturing regime can transform to a critical state and critical rupture may occur. We present the possibility of using these results in the operational reservoir to manage seismic hazards.

## Introduction

Knowledge of the time-dependent strength of rocks and the deformation due to deep underground fluid injections is crucial for assessing seismic hazards and related risks. Understanding the evolution of the fracturing process that enhances the permeability of rocks is also important for optimizing the exploitation of geo-resources.

Rocks fail due to the unstable coalescence of microfractures^1^. Initially, fracture growth is quasi-static, with small increases until the energy balance favours arrest of the rupture. However, fractures can interact due to further loading and nucleate a larger-scale fracture. Such fracture linkage may lead to the development of runaway instability under dynamic, critical conditions. Quasi-static fracture growth is one of the principal mechanisms of rock deformation and is subcritical^2,3^. Subcritical fracture growth (SFG) is time-dependent fracture growth at a value of the stress intensity factor that may be lower than the critical value of fracture toughness^2,4^. Several studies have discussed different aspects of the SFG mechanism in rocks, although they have mainly focused on tensile (mode I) loading. However, shear loading is more common in rocks; when accompanied by tension, it leads to mixed-mode fracture growth (modes I, II and III)^5^. Although subcritical shear fracture growth is rare, some studies have presented evidence of its occurrence^4^. The existence of SFG in shear mode loading has very important implications for the geological hazards associated with earthquakes, e.g., the time to failure, seismicity rate, magnitude distribution or maximum magnitude^2,3,6^.

The aims of this work are to assess the character of fracture growth during injection-induced seismicity and to determine how the technological loading conditions influence fracture growth. For this purpose, we focus on the seismicity observed at The Geysers (TG) geothermal field. Our considerations are based on the transition from the laboratory experiment scale to the field observation scale and are based on the following assumption. We consider injection-induced events as a series of local dynamic jumps in rupture length, localized along the rim of a larger-scale quasi-statically growing rupture. This distinction between single and systemized rupture was previously proposed by Main *et al*.^1^, who applied such logic to infer stress corrosion indices for SFG in laboratory compression experiments for a fractal ensemble of cracks. Small dynamic events within quasi-static fracture growth have also been studied in lab experiments by Lengliné *et al*.^7^. We analyse the rate of inferred incremental lengths associated with intermittent rupture propagation as a function of inferred underlying total rupture length. We relate changes in the scaling between the two to demonstrate an approximate power law relation between the two, consistent with Charles’ law for SFG in the case of slowly varying applied stress. Providing evidence for subcritical mixed-mode fracture growth at TG geothermal field, we evaluate the impact of the injection rate on SFG and on the magnitude of analysed earthquakes. We find that SFG is governed by the changes in stress due to the injection of water into the reservoir, and we provide the relation between the injection rate and the fracture growth rate. To infer the largest magnitude of the intermittent rupture network, we apply a theoretical scaling relation between the largest arrested magnitude of an earthquake and the injection volume developed by Galis *et al*.^8^ Since the SFG exponent provides insights into the fracturing process, we propose to modify the formula of Galis *et al*.^8^ by incorporating a parameter describing the rate of the fracturing process into the formula. We also discuss the stress state under which the fracture networks grow subcritically. We find that the subcritical rupture regime can be controlled by the stress parameter $R=({\sigma }_{1}-{\sigma }_{2})/({\sigma }_{1}-{\sigma }_{3}),$ where ${\sigma }_{i},\,i=1,2,3$, are the principal stresses. We find that if ${\sigma }_{2}$ is close to ${\sigma }_{1}$ or ${\sigma }_{3}$, the rate of SFG is statistically significantly lower than for the conditions when the principal stresses are more symmetrical, i.e., ${\sigma }_{2}$ is centred between ${\sigma }_{1}$ and ${\sigma }_{3}$. Finally, we analyse conditions under which the subcritical rupture regime can experience a transition to a critical one and critical rupture may occur.

## Data

We investigate the development of fractures using seismic data recorded in the northwestern part of TG geothermal field in California, USA, related to large-scale, long-term fluid injection into two wells, Prati-9 and Prati-29. TG is located in a series of complex, intensely deformed and faulted metamorphic Franciscan greywackes (e.g.,^9^). The seismicity in this region results from the thermoelastic and poroelastic effects that influence the local stress field in the vicinity of the injection wells (e.g.,^9–12^). The regional stress regime in the TG field is extensional. Based on the fault plane solutions of seismicity recorded in this area, a NNE orientation of the maximum horizontal stress is found, indicating a combined normal and strike-slip faulting regime, and the strike-slip component increases with reservoir depth^13^. The catalogue of 1254 seismic events and injection rate data is available on the induced seismicity European Plate Observing System platform of the EPOS Thematic Core Service Anthropogenic Hazards (IS-EPOS, https://tcs.ah-epos.eu). Seismic source parameters of these events were calculated by Martínez-Garzón *et al*.^9^ and Kwiatek *et al*.^10^ For the purpose of the following analysis, the stress drops are calculated based on seismograms using seismic moments and corner frequencies following^14^ (Materials and methods).

**Figure 1:**
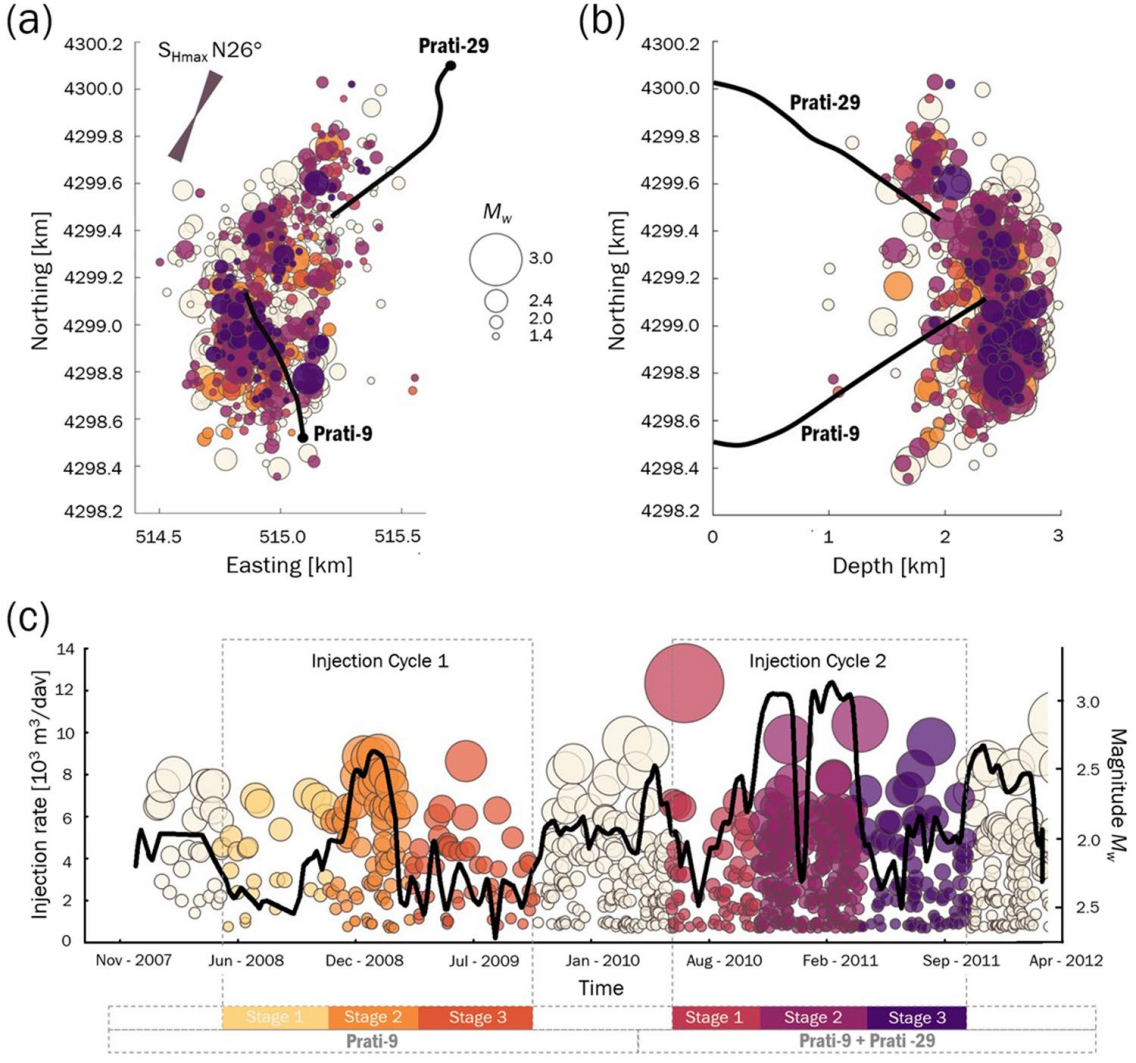
Hypocentre distribution of seismicity: (**a**) map view and (**b**) south-north section. (**c**) The daily injection data for Prati-9 and Prati-29 between November 2007 and April 2012. Yellow to purple colours indicate the stages of the two analysed injection cycles: 1- preceding, 2- during, and 3 – following peak injection.

We consider two of the injection cycles described in^9^. We select these cycles after^9^ since they frame the most intensive fluid injection into the reservoir, resulting in a large number of seismic events. In total, 509 seismic events occurred in the period between April 2008 and October 2011^10^. The cycles include peaks of fluid injection during two different periods, i.e., when only the Prati-9 well is active and when both wells Prati-9 and Prati-29 are operating. Moreover, detailed technological activity data during these periods are available in the literature (e.g.,^9^). Each cycle is divided into three stages: preceding, during, and following peak injection. The selected cycles exhibiting these stages are presented in Fig. 1, along with the daily injection data.

Our considerations of SFG refer to system-sized ruptures; thus, we use the fracture networks identified in TG by Orlecka-Sikora *et al*.^15^. Because the reservoir rocks in TG are highly fractured, the authors assumed that its fractures propagate along pre-existing fractures, which has also been suggested to be the dominant mode of failure at the crustal level^2^. Furthermore, the authors used seismic source parameters to identify similarly oriented fractures according to criteria based on the fault plane orientations of fractures and the locations of hypocentres with respect to the injection well and the regional principal stress orientation. These fractures, referred to here as a fracture network (FN), are assumed to represent the damage in the vicinity of the same system-sized ruptures. To identify fracture networks, these authors used hierarchical clustering. This approach resulted in the identification of 13 FN in the time period we are focused on here^15^.

## Results

In subcritical hydraulically driven fracture growth, a fracture grows due to either an increase in fluid pressure or a decrease in normal stress (e.g.,^16,17^). The long-term loading of pore fluid stresses and thermal stresses can weaken a rock’s resistance to fracture. These mechanisms have been shown to be responsible for the seismicity observed in TG (e.g.,^9,10,18^). Previous studies of seismic moment tensors in TG revealed a mixed-mode fracture mechanism^19–22^. Laboratory experiments have determined that the values of the parameters for mode II and mode III SFG are similar to the corresponding values of mode I SFG regardless of the loading configuration or specimen geometry^4,23^. Based on these results, we assume that the constitutive equations describing subcritical tensile crack growth hold for all three fundamental modes of loading (e.g.,^2,4,23^). The most widely used model is Charles’ power law^24^, which describes the crack tip velocity in a subcritical regime^2^. Charles’ law can be considered in the following form, which describes the rate at which a fracture grows:

1 $$v(t)=\frac{dl}{dt}=C\cdot {l}^{a},$$

where $l$ is the fracture length or diameter; $a$ is the growth exponent, which is related to the stress corrosion index $n$ by $a=n/2$, and $C$ is a parameter that depends on the stress state. The acceleration of the fracture length is predicted by solving Eq. (1):

2 $$l={l}_{0}{(1-\frac{t}{{t}_{f}})}^{2/(2-n)},$$

where ${l}_{0}$ is the initial fracture length at time $t=0$ and ${t}_{f}$ is the failure time^25^. In the case of a constant stress, instability can develop if $n > 2$. Therefore, if the average increase in fracture unit length over time is high, it would lead to sudden unstable fracture propagation, resulting in a runaway earthquake.

The fracture length is proportional to the cube root of the seismic moment, ${M}_{0}$^26^. The relation between$\,{M}_{0},\,$and the seismic source dimension, ${r}_{0},$ for the analysed dataset was provided by Kwiatek *et al*.^10^ The moment magnitude is calculated from ${M}_{0}\,$using the equation of Hanks and Kanamori^27^: ${M}_{w}=({\log }_{10}{M}_{0}-9.1)/1.5$. We focus on the fracture network growth rate. The criteria applied to identify fracture networks by Orlecka-Sikora *et al*.^15^ allow us to track the coseismic increases in fractures that occur during seismic events. In our considerations, rupturing due to seismic events builds a fracture network. Such an approach is in line with the observations of injection-induced seismicity and the results of laboratory experiments (e.g.,^28,29^). However, in the case of geothermal reservoirs, there is evidence for the importance of aseismic growth processes (e.g.,^30–33^). We assume that the ratio of seismic to aseismic growth is constant under certain stress conditions; thus, we take the total seismic length as a proxy for the total seismic and aseismic length. Hence, the complexity of fracture growth directly reflects the complexity of the developing network structure. We consider the growth rate for every FN identified by^15^ during each injection stage in both cycles. To reduce the data scatter, especially that in the injection rate loading data, when considering $\frac{dl}{dt}$, we use the observed average increase in FN length per unit time during a particular stage of injection as follows:

3 $${v}_{mean,\Delta t}(l)=\frac{{\sum }_{i=1}^{n}\,d{l}_{i}}{{\sum }_{i}^{n}\,d{t}_{i}}=C\cdot {l}^{a},$$

where $i=1,\ldots n$ is the number of observed increases in the FN in the $\Delta t$ time period due to constant loading, which is assumed using the mean injection rate ($mIR$) in $\Delta t$. The FN propagates with a mean velocity of ${v}_{mean}$ in $\Delta t$, and $l$ is the total length of the FN at time $t$. The SFG parameters $a$ and $C$ are determined based on the slope and intercept of the linear regression through all $\log ({v}_{mean,\Delta t})$ values versus $\log \,(l)$ values.

### Evidence for SFG

**Figure 2:**
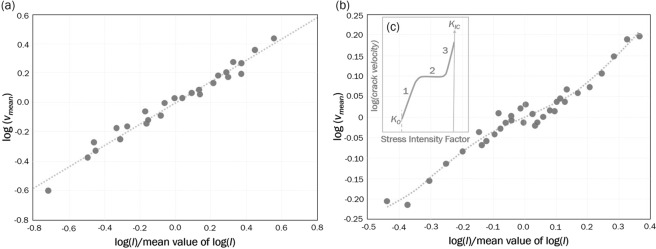
Characteristic patterns of the proxy for the crack velocity/normalized stress intensity diagram for SFG in TG. (**a**) Data represent growth of 3 fracture networks during 2 stages preceding and 1 stage following the peak injection in cycle I; (**b**) Data represent growth of 2 fracture networks during the stage of peak injection in cycle II; (**c**) Schematic relation between the stress intensity factor and crack velocity for subcritical tensile crack growth; *K*_*IC*_ is the critical stress intensity factor, *K*_0_ is the stress corrosion crack growth limit, and 1–3 represent different behaviours of SFG.

We observe two types of patterns for $\log (v)-\,\log (l)$ dependency; the most common one is the linear pattern, and the second pattern is close to the experimentally derived characteristic $K-v$ diagram for tensile SFG, which has three distinct regions of $K-v$ dependency^2^, where $K$ is the stress intensity factor (Fig. 2c). Generally, the stress intensity factor at the crack tip is proportional to the applied stress and the square root of the fracture length (Eq. (3))^4,34,35^. The complete $K-v$ diagram for tensile SFG derived from studies of glass is assumed to hold for all three fundamental modes of crack displacement, although there is little evidence to support this assumption^2^. The behaviour in region 1 (the area marked as 1 in Fig. 2c) is assumed to be controlled by the rate of stress corrosion reactions at the crack tips. Region 2 (marked as 2 in Fig. 2c) is controlled by the rate of transport of reactive species to the crack tips. In region 3 (marked as 3 in Fig. 2c), crack growth is mainly controlled by mechanical rupture^2^. Most experimental data obtained from studies of tensile SFG in rocks appear in region 1 or region 3 on the $K-v$ diagram. Region 2, which corresponds to the diffusion process, is very rarely observed in rocks^2^.

**Figure 3:**
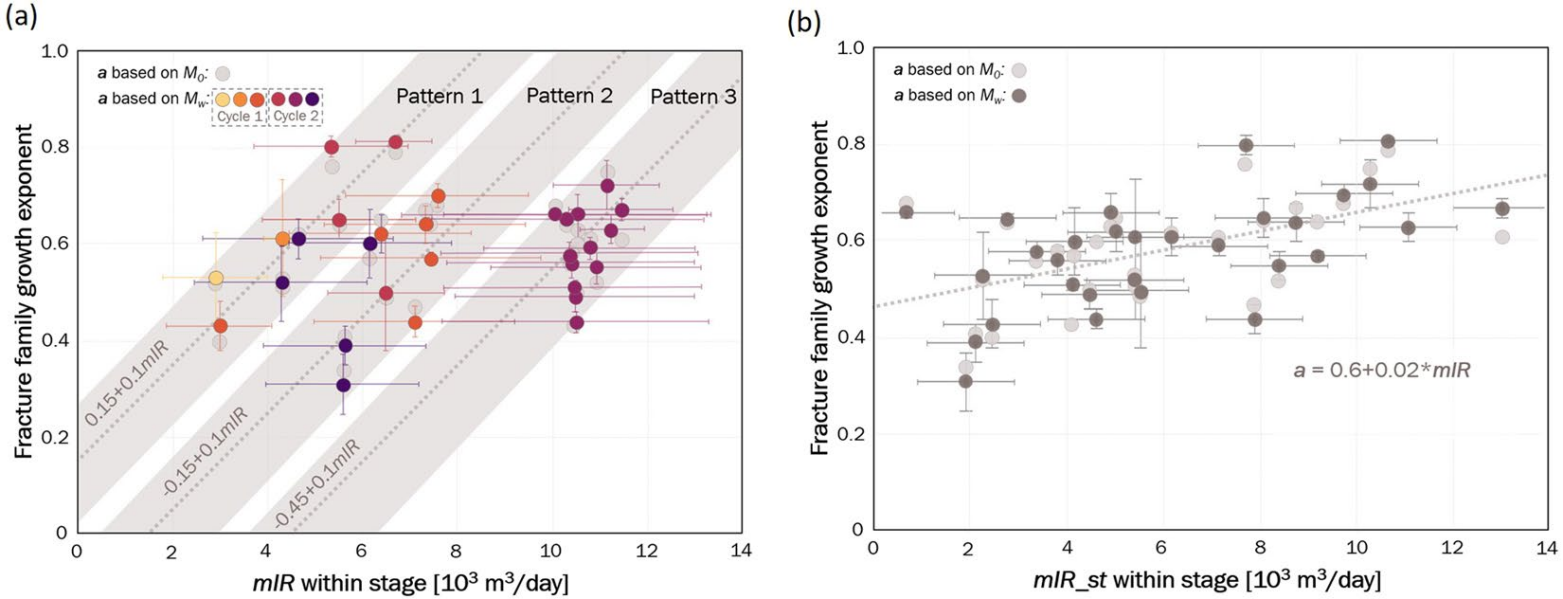
Subcritical fracture network growth exponent versus (**a**) $mIR$ in each injection stage and (**b**) standardized $mIR$ in each injection stage in the $mIR$ intervals denoted by Patterns 1–3 in (**a**). The horizontal and vertical bars denote the standard deviations of $mIR$ and $a$ parameter estimates, respectively.

Of the 66 fracture network growth periods associated with the stages of a particular injection cycle, we can estimate the values of the growth exponent $a$ and the parameter $C$ for 33 cases. Information about the uncertainties of these estimates is provided in the Materials and methods section. Although the standard errors of estimation ($SEEs$) of the $a$ and *C* parameters are very low for most of the injection stages, we observe variations in the $a$ and $C$ parameters related to the injection rate (Fig. 3). The results reveal a cyclic-like pattern in the relation between the growth exponent, $a$, and the mean injection rate, $mIR,$ in the stage. In the first range of $mIR$ values (Pattern 1 in Fig. 3), which reach up to approximately $7\cdot {10}^{3}\,{{\rm{m}}}^{3}/{\rm{day}}$, the relation between the injection rate and growth exponent is $a=0.15+0.1\cdot mIR$. The Spearman correlation coefficient between the injection rate and growth exponent is 0.9 and is statistically significant. Then, at injection rates ranging from $4.5\mbox{--}7.5\cdot {10}^{3}\,{{\rm{m}}}^{3}/{\rm{day}}$ (Pattern 2 in Fig. 3), the relation is repeated with higher scatter, with a statistically significant correlation coefficient of 0.7. When $mIR$ exceeds $8.0\cdot {10}^{3}\,{{\rm{m}}}^{3}/{\rm{day}}$ (Pattern 3 in Fig. 3), we observe a relation in $mIR$ versus $a$ with a statistically insignificant correlation coefficient of 0.3. When $mIR$ is standardized within Patterns 1 to 3, the relation of $mIR$ and $a$ is statistically significant with a correlation coefficient of 0.5; the value of $a$ is equal to approximately $0.6$, with a standard deviation of 0.02, and the value of $C=0.02$, with a standard deviation of 0.006. Although the values of the $C$ parameter range from $2\cdot {10}^{-3}\mbox{--}2\cdot {10}^{-2}$, they generally decrease with $mIR$.

### Seismic potential of SFG in TG – the maximum subcritical magnitude

**Figure 4:**
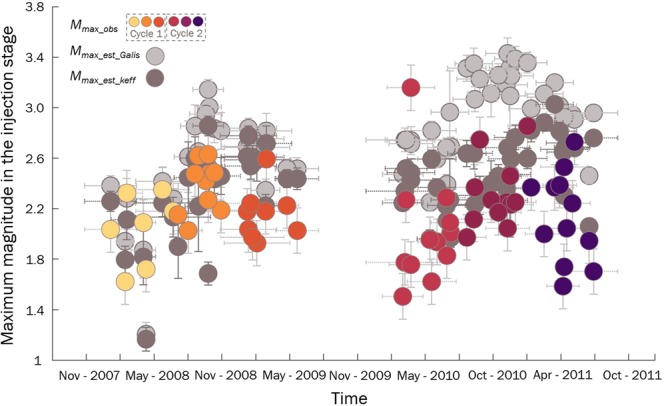
Maximum moment magnitude, ${M}_{w}^{max},\,$versus time in the injection stages. Yellow to purple colour dots are the observed ${M}_{w}^{max}$ in the stages of injection cycles; light grey dots are the ${M}_{w}^{max}$ estimated from the Galis *et al*.^7^ formula; dark grey dots are the ${M}_{w}^{max}$ estimated with the modified bulk modulus. The horizontal and vertical bars correspond to the estimated standard deviations of the variables. The Spearman correlation coefficient for observed ${M}_{w}^{max}$ and $mIR$ is equal to 0.4 and is statistically significant.

Since injection-induced seismicity in TG provides evidence for SFG, we analyse its seismic potential from the model of the maximum magnitude of self-arrested rupture, ${M}_{w}^{max-arr}$, proposed by Galis *et al*.^8^ We apply this model to each step of FN growth due to an increase in the injection rate. According to^8^, the maximum arrested seismic moment, ${M}_{0}^{max-arr}$, and the corresponding ${M}_{w}^{max-arr}$ can be estimated by approximating the additional stress drop induced by the perturbation of pore pressure as a point load superimposed on the background stress drop (i.e., the stress drop before injection), $\varDelta {\sigma }_{0}$. This approximation yields the dependence of earthquake size on the injection volume, $\varDelta V$, with an exponent of 3/2^8^:

4 $${M}_{0}^{max-arr}=\gamma \varDelta {V}^{\frac{3}{2}},\,{\rm{where}}\,\gamma =\frac{0.4255}{\sqrt{\varDelta {\sigma }_{0}}}\cdot {(\frac{\kappa \varDelta {\mu }_{d}}{h})}^{\frac{3}{2}}$$

where $\kappa$ is the bulk modulus of the reservoir rock, ${\mu }_{d}$ is the dynamic friction coefficient of the fault and $h$ is the reservoir thickness. In this model, we use parameter values that are plausible for TG: the bulk modulus of the fracture-dominated reservoir rocks $k=3.6\cdot {10}^{9}Pa$, Poisson’s ratio $\nu \,=\,0.25$, $h=1500\,m$, and ${\mu }_{d}=0.1$^9,36^. We assume after Galis *et al*.^8^ that earthquake stress drops reflect the background stress drop, i.e., the stress drop before the following injection loading, $\varDelta {\sigma }_{0}$. Hence, we apply the mean $\varDelta {\sigma }_{0}$ in each injection stage, following the stage for which ${M}_{w}^{max-arr}$ is calculated. The volume of injected water is calculated based on $mIR$ and the duration of the stage in a particular FN. Generally, the temporal evolution of the maximum observed magnitude follows the behaviour predicted by the model of Galis *et al*.^8^ (Fig. 4). However, the difference between the upper limit predicted by^8^ and the observed maximum magnitude increases with time. During the first cycle of water injection, the observed maximum magnitudes for the particular stages of fracture networks correspond well to the estimated ${M}_{w}^{max-arr}$. However, during cycle 2, the observed maximum magnitudes are much lower than the arrested model estimates. This observation is not unique; the same trend can be observed for the recorded maximum magnitudes during a 6.1-km-deep geothermal stimulation in Finland^37^. Previous studies of the deformations and seismicity in TG showed that the bulk modulus varies in a wide range (e.g.,^36^). The variability of the bulk modulus for the reservoir at TG can be associated with the fracture density and is lower for more fractured rocks (e.g.,^36^) and higher when pressure increases (e.g.,^38^). Since the SFG exponent provides insights into the fracturing process, we propose to modify the formula of Galis *et al*.^8^ by incorporating the $a$ parameter to estimate the effective bulk modulus for TG: the higher the $a$ value is, the lower the $k$ modulus. Based on the well-confirmed functional dependence of the rock bulk modulus on crack density (e.g.,^38^), we use a simple coefficient $k=(1-a)\cdot 3.6\cdot {10}^{9}Pa$ to obtain the effective value of the bulk modulus for the rocks experiencing a particular FN. Because the relation $mIR-a$ is shifted with $mIR$ for Patterns 2 and 3 (Fig. 3), which is linked to the pressure changes because the fracture density increases, the $(1-a)$ coefficient is reduced by (1-shift). Figure 4 presents the maximum magnitudes observed during a particular stage of water injection together with the estimated values of ${M}_{w}^{max-arr}$ from the seismic moment according to Eq. (4) and with the modified bulk modulus. The differences between the observed maximum magnitudes and upper limit derived from Galis *et al*.,^8^ expressed by the root mean square error (*RMSE*) is 0.7, and for the modified equation, by incorporating the subcriticality to the Galis *et al*.^8^ model, the *RMSE* is 0.5. The estimated ${M}_{w}^{max-arr}$ for the period framed by both injection cycles, without the distinction to the cycles and stages, is 4.0.

### The instability

**Figure 5:**
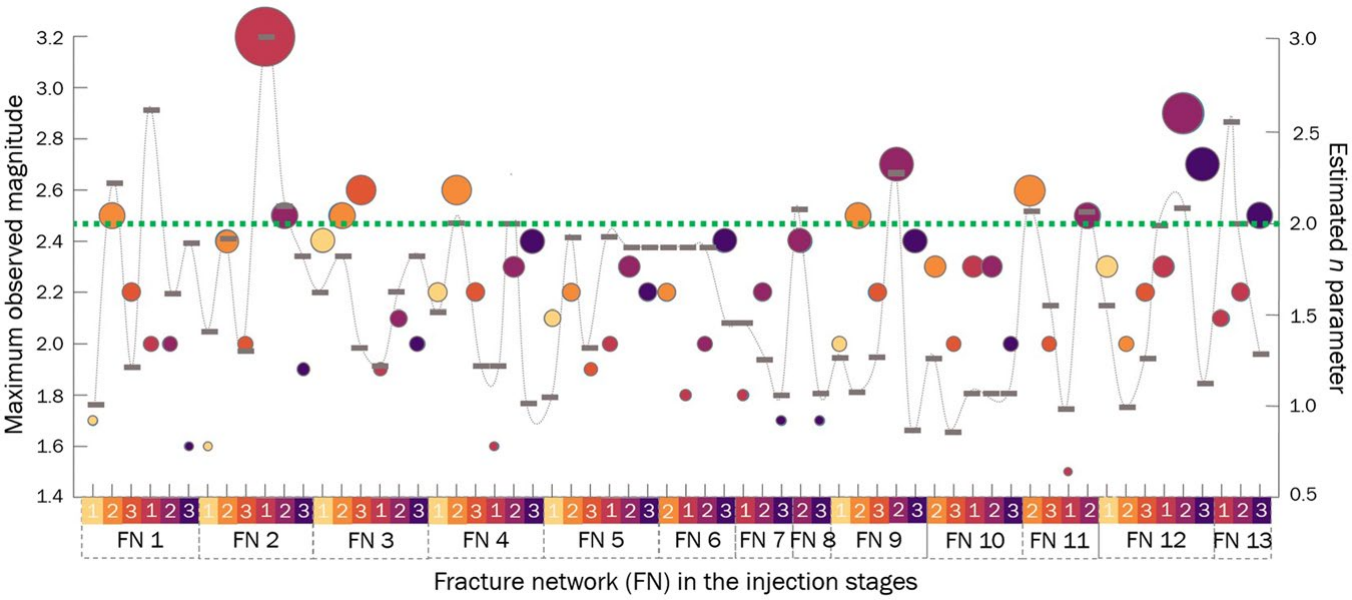
Changes in the stress corrosion index $n$ for the fracture network growth stages, with the strongest earthquakes observed in each stage. The circles, colour-coded by injection stage, are the observed maximum moment magnitudes with their sizes proportional to magnitude. The horizontal bars denote the stress corrosion index of the FN stage. Instability is expected for FN stages with the stress corrosion index $n\, > 2$, i.e., above the green horizontal line $n=2$.

A fracture growing subcritically can reach instability and become the critical fracture propagating dynamically. The instability requires a stress corrosion index $n > 2$. In our studies, the $a$ parameter is equal to the stress corrosion index $n\,$divided by 2. Moreover, our modified approach of tracking changes in the mean velocity of fracture network growth instead of using absolute velocity data (Eq. (3)) influences the $a$ value (Materials and methods) by approximately 30%. After recalculating $a\,$values to the stress corrosion index $n\,$and correcting for the mean velocity approach, we find some stages of fracture networks growing under $n\, > \,2$ (Fig. 5). Figure 5 presents the instabilities together with the strongest earthquakes recorded in the considered dataset. The occurrences of the strongest earthquakes in the stages are well correlated with the occurrences of instabilities. Fracture network 1 during the 3^rd^ stage of the 2^nd^ injection cycle experienced growth acceleration, but the strong event with ${M}_{w}\,2.5$ occurred 3 months later, after the analysed time frame of the 2^nd^ cycle. The U Mann-Whitney test, which tests whether two independent samples were selected from populations having the same distribution, confirms that the fracture networks with approximated values of parameter $n$ > 1.6 produce higher maximum observed magnitudes than the FN stages with lower $n$, with the *Z* statistics value of the test equal to 2.6 for a sample size of 15 for both groups, at a significance level $p=0.009$.

### Stress changes influencing SFG in TG

**Figure 6:**
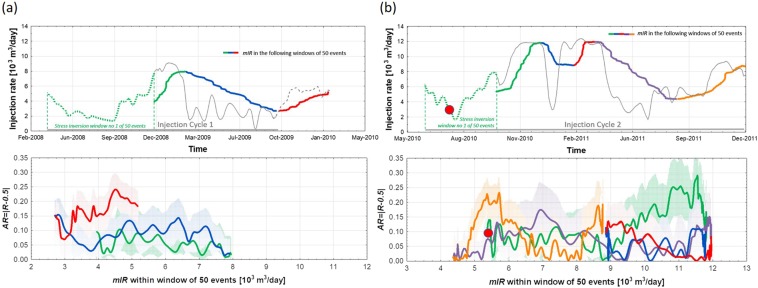
The absolute value of the difference between the stress magnitude *R* and a value of 0.5 for the dataset without distinguishing between FNs versus $mIR$ during (**a**) cycle 1 and (**b**) cycle 2. For the entire dataset, the relative stress magnitude *R* is calculated using moving windows from 50 earthquakes, with a step size of 1 event. Bars represent the uncertainties in *AR* estimates. The red dot indicates the strongest earthquake with ${M}_{w}=3.2$.

Thermo- and poro-elastic stresses are mainly responsible for the seismicity observed in TG. However, both mechanisms can act at the same time to decelerate or accelerate fracture growth^1^. These mechanisms can thus increase the crack density, leading to an increase in the growth parameter $a$ due to crack-linking processes that facilitate fracture extension. Alternatively, they can also lead to a decrease in microcrack density, thus inhibiting SFG. To infer the ability of rocks to develop microcracks, we focus on stress symmetry breaking. The degree of stress symmetry breaking yields information about how far the value of the intermediate stress (${\sigma }_{2}$) is from the midpoint between the values of the maximum principal stress (${\sigma }_{1}$) and the minimum principal stress (${\sigma }_{3}$). The results of laboratory experimental studies show that the effect of dilatancy is influenced by the intermediate principal stress (e.g.,^39,40^). If the intermediate stress is close to ${\sigma }_{1}$ or ${\sigma }_{3}$, the dilatancy increases. However, if the principal stresses approach symmetry, i.e., ${\sigma }_{2}$ is centred between s1 and ${\sigma }_{3}$, the rock is strengthened, leading to a significant weakening in the effect of dilatancy. For this purpose, we calculate the relative stress magnitude $R=\frac{{\sigma }_{1}-{\sigma }_{2}}{{\sigma }_{1}-{\sigma }_{3}}$ following the stress inversion methodology used in the STRESSINVERSE software package^41^. STRESSINVERSE calculates the stress orientation based on focal mechanisms (strike/dip direction/dip angle). The inversion is based on Michael’s method^42,43^, in which an instability criterion proposed by Lund and Slunga^44^ is incorporated. We use 1000 noise samplings of the input focal mechanisms to estimate the 95% confidence intervals of the relative stress magnitude. We perform a temporal analysis of the stress field changes and assess the impact of depth on the stress inversion results. For the temporal analysis, we apply the stress inversion to the entire dataset, i.e., without dividing the data based on the fracture networks due to the limited number of seismic events in the stages of the FNs. Then, we perform a stress inversion of the focal mechanisms from the moving windows of 50 earthquakes using a step size of 1 event to detect small variations in the relative stress magnitude. The number of events in the moving window is selected to achieve balance via a trade-off between the discrimination of different injection rates and the requirement of a certain variety of focal mechanisms. The rock strength is expected to be highest at $R=0.5$ and lowest at $R=0$ and $R=1$. Hence, we analyse the absolute value of this difference $AR=|R-0.5|$ as a measure of the amount of stress symmetry breaking, with $AR=0$ representing the maximum rock strength. Although the $R\,$parameter is strongly uncertain, its relative changes are plotted versus $mIR$ (Fig. 6). We observe both rock strengthening and rock weakening when decreasing and increasing the injection rate.

**Figure 7:**
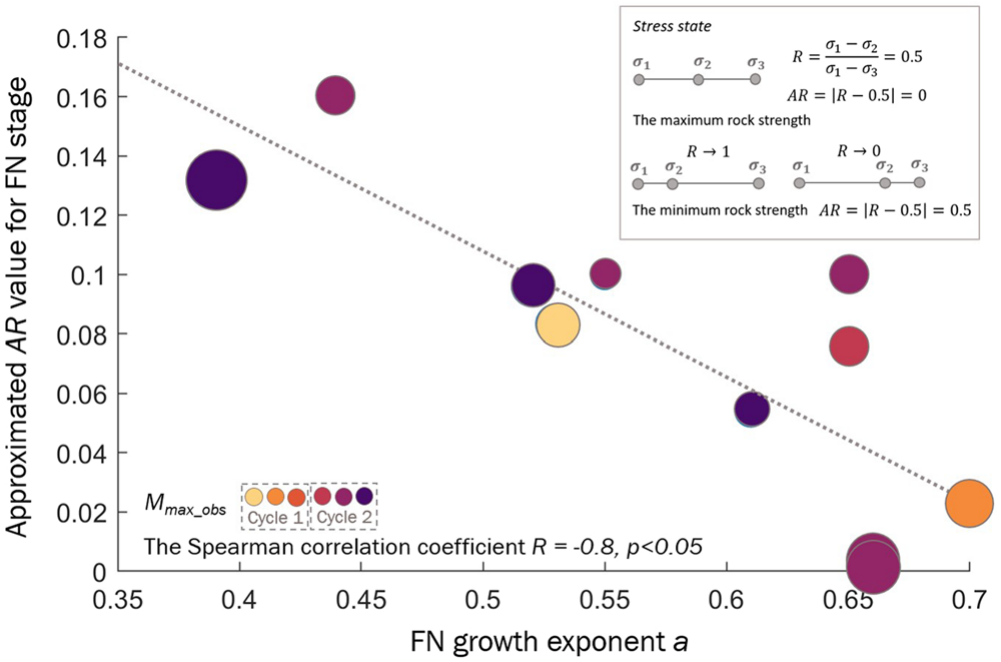
The fracture network growth parameter $a$ versus $AR$ values estimated for the consecutive 50-event windows with overlaps of 10 events for the analysed dataset. The circles, colour-coded with injection stages, are the observed maximum moment magnitudes with their sizes proportional to magnitudes from 2.1 to 2.7.

Since the $AR$ value is calculated for the entire dataset in a moving window in time, directly assessing the relation between the $a$ parameter and $AR$ for the FN stages is difficult. To enable this determination, we select in each window the most frequently occurring FN stage as most representative of the stress state during the period framed by the window, and we associate with this window the $a$ parameter of this FN stage. To overcome the possible influence of the overlapping window approach on the statistical inference, we reduce the range of overlap to a maximum of 10 events since the number of nonoverlapping windows is too small to perform the analysis. In this case, the entire series of 509 events is used to construct 12 consecutive windows of 50 elements each with overlaps of 10 events. The statistical test confirms that higher values of the $a$ parameter are associated with lower values of $AR$, while the FN stages with $a$ <0.6 experience higher $AR$, with the *Z* statistics value of the U Mann-Whitney test equal to 2.1, at a significance level $p=0.035$. Moreover, there is a significant correlation between the $a$ parameter and $AR$ for the tested windows equal to -0.8 (Fig. 7).

## Discussion and Conclusions

The plot of the mean inferred change in fracture length per unit time versus length implies that the fracturing process in TG is controlled by SFG, with some episodes of instability. The FN growth rate depends on the FN length and the stress parameter. The mean value of parameter$\,a$ is approximately $0.8$ after correction, and the average value of the stress parameter $C$ is approximately $0.1\,m/day$ $[0.1\cdot {10}^{-5}\frac{{\rm{m}}}{{\rm{s}}}]$ when the fracture length is approximated by the cube root of ${M}_{0}$. This value corresponds to the flow velocities estimated for cycle 1 and cycle 2. The mean flow velocities for the reservoir with a radius of $300\,m$, i.e., at the shortest distance from the injection well where seismicity occurred under the assumption of $0.24\,M{m}^{3}/month$ for cycle 1 and $0.32\,M{m}^{3}/month$^9^ are $0.04\,m/day$ for cycle 1 and $0.1\,m/day$ for cycle 2.

Subcritical properties vary with fluid injection loading, and fracture growth occurs with the $a$ parameter ranging from $0.4$ to $1.3$ after correction. At high injection rates, the $a$ parameter is usually high, and at low to mean injection rates, the $a$ parameter varies from low to high values. The values of the fracture growth parameter obtained here are lower than those derived from laboratory tests. Atkinson *et al*.^2^ provided an extensive list of the estimated fracture growth index values for Charles’ law during laboratory tests. The values of this parameter are found to range from 12 for quartz rocks under water vapor and a temperature of 200 °C to approximately 40 for calcite, basic, granitic and sedimentary rocks and to much higher values for basalts. However, fracture growth index values have also been obtained for geological faults at the crustal scale; they are much lower than those derived from laboratory tests and rarely exceed 4^45^. In our studies, the stress corrosion indices ranges from $0.8$ to $2.6$. These results are similar to those derived for rock mechanical experiments interpreted using the mean field theory for subcritical crack growth based on a modified Griffith criterion for a fractal ensemble of cracks, which range from 0.7 to 3.5^1^. We find that the velocities of fracture growth are much smaller than the seismic rupture propagation. The fracture growth velocity in TG ranges from ${10}^{1}$ to ${10}^{2}$ $\frac{{\rm{m}}}{{\rm{s}}}$, which corresponds to the hydraulic diffusivity in the region of TG on which we focus (e.g.,^9^). This is clear evidence of quasi-static SFG on the system scale, i.e., by intermittent dynamic (seismic) fracture.

Our analysis shows that the proposed scaling for the arrested maximum magnitude with the injected volume of Galis *et al*.^8^ provides good estimates when the injected volume is not high. The temporal evolution of seismicity in TG shows that the observed maximum magnitudes increase the time to the estimated ${M}_{w}^{max-arr}$ when the injected volume increases. After incorporating the $a$ parameter to estimate the effective bulk modulus for TG in the Galis *et al*.^8^ formula, the maximum observed subcritical magnitudes adequately fit the predicted ${M}_{w}^{max-arr}$ over the whole time. We relate this behaviour to stress conditions favouring the occurrence of fractures that are randomly distributed in the rock volume and thus decreasing their linkage potential. These conclusions are supported by the results of the analysis of the connectivity process during water injection into the reservoir in TG provided by Orlecka-Sikora *et al*.^15^ and Lasocki and Orlecka-Sikora^46^. The statistical tests confirmed that lower injection rates favoured linking fractures, whereas higher injection rates inhibited linking^15^. This is also in line with the results of our present analysis of the relation between the fracture growth rate and the stress symmetry. Fjaer and Ruisten^40^ explained the observed dependency of the rock strength and reduced stress symmetry by linking rock heterogeneity to the impact of the intermediate principal stress. This effect was modelled macroscopically by assigning a smoothened Coulomb failure criterion to each possible orientation of a failure plane in a rock and combining the failure probability of each plane into the overall probability of the failure of the rock. Based on their results, which were obtained at low $AR$ values, only two potential orientations of the failure plane fulfil the Coulomb failure criterion; as a consequence, the expected value of rock strength is higher^40^. We conclude that at lower $AR$ values, the rupturing process may lead to the occurrence of a runaway (critical) rupture because the potential background stress drop of the fault increases. During the analysed period in TG, the strongest seismic event with magnitude Mw 3.2 occurred during the first stage of the second cycle at a $mIR$ value of approximately $6.0\cdot {10}^{3}\,{{\rm{m}}}^{3}/{\rm{day}}$, where the $AR\,$value was low (Fig. 6). The estimated $a$ parameter obtained from the relation presented here at this $mIR$ value is 0.8 (the stress corrosion index exceeds 2).

According to the studies of Fjaer and Ruisten^40^, at high $AR\,$values, many orientations of the failure plane are equivalent once the Coulomb failure criterion is fulfilled, and the failure plane assumes the orientation at which the rock fails most easily, e.g., along some rock heterogeneities. This result indicates that fractures may be isolated with low stress drops due to weak rock conditions and that the fracture growth rate decelerates.

Our approach provides a new perspective on earthquake mechanics driven by fluid injection. Recognizing the phenomenon of SFG and its reaction to technological activity and combining this information with the characteristics of the stress state in the reservoir can be used to help manage seismic hazards and optimize technological production. Based on the SFG information, we may conclude that, e.g., $n < 2$ would imply the transient FN growth rate is slowing^26^, whereas $n > 2\,$would imply increasing event rate and risk of large events during operations. The analysis shows that a higher FN growth rate is related to a lower $B$ value in the Gutenberg-Richter relation (Materials and methods, The relation between *b* and the fracture growth rate $a$ parameter). This relation can also occur for exponential growth in the expected rate of seismicity with respect to stress or time, as in the Groningen gas field^26,47^, where there have been major interventions in managing the field. The rate of SFG and its relation to the technological stress change factor, which in this case is the water injection rate, provides information about the technological activities that can maintain stable fracture growth, far from a catastrophic runaway earthquake. To better monitor magnitude of seismic events the stress drops and injection information is needed. Seismic hazard management during fluid injection can then be supported by adjusting the injection schedule to keep the fluid pressure and the injected volume below a critical level, at which the observed maximum magnitude approaches the estimated arrested volume at the level set by the local authorities (e.g.,^37^). This insight into the time- and technology-dependent behaviour of rock also helps to identify the stress conditions under which time may be important for the stability of the rock structure, e.g., in mines.

## Materials and Methods

### Stress drop estimation

**Figure 8:**
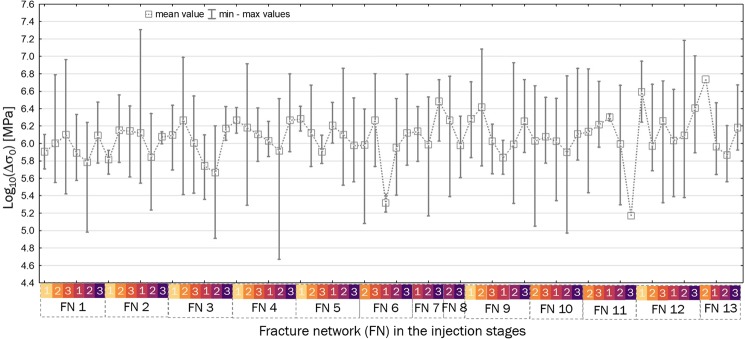
The range of the estimated values of logarithm of earthquake stress drops in the stages of fracture network growth. Yellow to purple colours indicate the stages of the two analysed injection cycles: 1- preceding, 2- during, and 3 – following peak injection.

Static stress drops were recalculated for the entire input catalogue following the methodology described in^9,10^. Therefore, we provide only a brief description of the source parameter assessment here. The 3-component seismograms obtained from stations located <20 km from the source of seismicity were instrument-corrected and filtered using a 0.2 Hz high-pass Butterworth filter. The 0.5-second time windows with waveforms containing direct P- and S-wave arrivals were tapered using 0.1 s von Hann’s taper. The ground P- and S-wave displacement spectra were calculated using the multitaper method^48^ separately for each sensor and component. The spectra from each station were then combined^9^. Each spectrum was then fitted separately to the Boatwright point source model, including the frequency-independent attenuation term^9^. The inversion problem, which relies on fitting the curve parameterized by the seismic moment, corner frequency, and quality factor, was optimized by a combination of grid search and simplex techniques. We calculated the median seismic moment and corner frequency for all stations fulfilling the quality criteria. The corresponding source radius was calculated assuming the circular source model of Madariaga^49^ and a standard rupture velocity of 0.9 ${V}_{S}$. Finally, the static stress drop was calculated following Eshelby’s formula^14^. Figure 8 presents the range of the earthquake stress drops in the stages of FN growth and $a$ parameter.

### Fracture growth parameter assessment

Table 1 contains the information about the estimates obtained here, together with the standard errors of the growth exponent $a$ and parameter $C$ for 33 of the 66 FN growth periods associated with the three stages of the two considered injection cycles described in the Results section.

**Table 1:** The uncertainties in the SFG parameters.

Fracture network	Injection cycle	Injection cycle stage: p -proceeding, c-containing & f -following peak injection	Determination coefficient ${{\boldsymbol{R}}}^{2}$	${\boldsymbol{a}}$	Standard error of ${\boldsymbol{a}}$	${\boldsymbol{logC}}$	Standard error of ${\boldsymbol{logC}}$	Number of observations	Standard error of estimation	*p* value of ${\boldsymbol{a}}$
1	I	c	0.99	0.70	0.03	−5.03	0.09	11	0.02	0
	II	p	0.99	0.81	0.03	−6.12	0.14	8	0.003	0
		c	0.95	0.51	0.03	−4.82	0.14	17	0.01	0
2	I	c	0.97	0.62	0.04	−4.83	0.12	8	0.03	0
	II	c	0.98	0.67	0.02	−5.64	0.09	19	0.01	0
		f	0.97	0.60	0.07	−5.47	0.31	4	0.003	0.013
3	I	f	0.88	0.38	0.07	−3.96	0.85	6	0.01	0.006
	II	c	0.95	0.55	0.03	−4.99	0.15	18	0.01	0
4	I	c	0.97	0.64	0.04	−4.76	0.13	9	0.03	0
		f	0.96	0.43	0.05	−4.26	0.17	6	0.01	0.001
	II	c	1.0	0.65	0.01	−5.5	0.04	20	0.005	0
		f	0.78	0.31	0.06	−4.01	0.27	10	0.009	0.001
5	I	c	0.92	0.61	0.18	−4.83	0.4	3	0.04	0
	II	p	0.52	0.35	0.33	−4.49	1.18	3	0.023	0.48
		c	0.98	0.56	0.03	−5.33	0.11	11	0.01	0
		f	0.99	0.61	0.04	−5.52	0.15	11	0.01	0
6	I	c	1.0	0.57	0.01	−4.72	0.02	3	0.002	0.011
	II	c	0.99	0.58	0.02	−5.47	0.06	9	0.006	0
		f	0.89	0.52	0.07	−5.20	0.27	9	0.02	0
7	II	p	0.89	0.50	0.12	−4.31	0.36	4	0.02	0.054
		c	0.95	0.49	0.03	−4.29	0.1	18	0.03	0
8	II	c	0.96	0.66	0.04	−5.04	0.13	13	0.05	0
9	I	c	0.96	0.44	0.03	−3.92	0.1	9	0.02	0
	II	c	0.99	0.72	0.02	−5.69	0.09	8	0.003	0
10	I	c	0.98	0.76	0.05	−5.19	0.11	7	0.06	0
	II	c	0.99	0.56	0.02	−4.98	0.08	21	0.01	0
11	II	c	0.99	0.66	0.01	−5.19	0.05	24	0.01	0
12	I	c	0.99	0.34	0.02	−3.81	0.07	4	0.01	0.005
	II	p	0.79	0.37	0.06	−4.16	0.26	11	0.02	0
		c	0.99	0.59	0.12	−5.13	0.08	19	0.01	0
		f	0.92	0.39	0.04	−4.23	0.18	11	0.01	0
13	II	p	1.0	0.8	0.02	−5.9	0.04	10	0.02	0
		c	0.99	0.63	0.03	−5.39	0.08	12	0.02	0

The results of the linear regression analysis for the logarithm of the mean velocity of the fracture network growth and logarithm of the total length of the fracture network.

Both analysed variables, i.e., the logarithm of the total length of the FN, which is expressed by the cumulative moment magnitude of seismic events, and the logarithm of the mean velocity of FN growth, which is expressed by the cumulative moment magnitude of seismic events divided by the cumulative interevent time, are linearly dependent during the considered stages of water injection, as presented in Table 1. The estimated values of the FN growth parameter $a$ are significant in almost all FN growth stages because their *p*-values are below the significance level of 0.05. The only two exceptions are the growth parameter $a$ value obtained in the FN marked as 7, which has a *p* value of 0.054 during the stage proceeding the peak injection in the second cycle of water injection and that in the FN marked as 5, which has a *p* value of 0.48 during the stage containing the peak injection in the first cycle of injection. The determination coefficient, ${R}^{2}$, i.e., the proportion of the variance in the dependent variable that can be predicted from the independent variable, is very high, indicating that the regression models built on the total length of the FN explain almost all of the variance in the mean velocity of FN growth in the water injection stages considered. The values of standard error in the regression models are also very low, which indicates the high precision of the model’s predictions. In all other stages of water injection cycles, which are not presented in Table 2, we do not observe any linear dependency between the considered variables.

**Table 2:** The results of the estimation of the fracture network growth parameter $a\,$using the modified model of fracture network growth given by equation^3^.

Assumed value of parameter ${\boldsymbol{a}}$	Modified model of the velocity of fracture network growth						
	Estimated value of parameter $a$ according to Eq. (3) and the standard deviation of the estimate, ${\boldsymbol{std}}(\hat{{\boldsymbol{a}}})s$						
	*n* = 10	*n* = 20	*n* = 30	*n* = 50	*n* = 100	*n* = 130	*n* = 150
0.3	0.21, 0.002	0.22, 0.001	0.22, 0.001	0.23, 0.001	0.24, $4\cdot {10}^{-4}$	0.24, $4\cdot {10}^{-4}$	0.24, $4\cdot {10}^{-4}$
0.4	0.28, 0.003	0.29, 0.002	0.29, 0.002	0.3, 0.001	0.31, 0.001	0.31, 0.001	0.32, 0.001
0.5	0.34, 0.004	0.35, 0.003	0.36, 0.002	0.37, 0.002	0.38, 0.001	0.39, 0.001	0.39, 0.001
0.6	0.41, 0.01	0.42, 0.004	0.43, 0.003	0.44, 0.002	0.45, 0.001	0.45, 0.001	0.46, 0.001
0.7	0.47, 0.01	0.48, 0.01	0.49, 0.004	0.5, 0.003	0.51, 0.002	0.52, 0.002	0.52, 0.002
0.8	0.53, 0.01	0.54, 0.01	0.55, 0.005	0.56, 0.004	0.57, 0.003	0.58, 0.002	0.58, 0.002
0.9	0.58, 0.012	0.59, 0.01	0.60, 0.006	0.62, 0.005	0.63, 0.004	0.64, 0.003	0.64, 0.003
1.0	0.64, 0.014	0.65, 0.01	0.66, 0.008	0.67, 0.007	0.68, 0.005	0.69, 0.004	0.69, 0.004
1.1	0.69, 0.017	0.70, 0.012	0.71, 0.01	0.72, 0.008	0.73, 0.006	0.74, 0.006	0.74, 0.006
1.2	0.74, 0.021	0.75, 0.015	0.76, 0.012	0.77, 0.01	0.78, 0.008	0.78, 0.008	0.79, 0.007
1.3	0.79, 0.024	0.79, 0.018	0.80, 0.015	0.81, 0.01	0.82, 0.01	0.83, 0.009	0.83, 0.009

The input datasets used for the assessment of the modified model performance are the moment magnitudes of the identified fracture networks in The Geysers and the interevent times calculated using the assumed fracture growth parameter $a$ in Charles’ law given by^2^; *n* is the size of the dataset.

### The differences in SFG parameters obtained when assuming the mean velocity of fracture network growth instead of actual velocity in the SFG model

Here, we assume the dependency of fracture growth velocity on fracture length, as described by^2^. For this purpose, we use the observed magnitudes in all identified FNs to calculate the expected velocities of fracture growth for values of the growth parameter $a$ ranging from 0.3 to 1.3. Then, for a given dataset, we calculate the parameter $a$ following the modified equation given by^3^. We then assess how the proposed approach influences the $a$ value and how this impact is related to the number of observations in the dataset, i.e., the number of segments in the FN. Table 2 presents the results of this analysis.

The modified model of FN growth influences the value of the parameter $a$. We observe that using lower real values of $a$ leads to a smaller underestimation of $a$. At the lowest considered value of $a=0.3$, the underestimation is 12%, while at the highest value of $a=1.3$, the underestimation reaches 37% of the $a$ value. In light of these results, we expect that the $a$ parameter of the FNs in TG, which varies from 0.31 to 0.81, is underestimated and that its real value is higher than that estimated by the FN growth model^2^. The results presented in Table 2 also show the influence of the number of seismic events in the dataset on the estimated results. In Table 2, we present the results obtained using *n* values corresponding to the number of seismic events in the growth stages of the identified FNs, which are usually less than 30, and those in the entire FN, which are usually less than 100. However, we also studied much higher values of *n* up to 5000 seismic events in the dataset. Within the range of $n$ values identified in the fracture arrays of the networks in TG, the estimated values of $a$ may vary up to ±0.03. These variations are much lower than the observed variations in the calculated$\,a$ parameter corresponding to the changes in the mean injection rate. Even at much larger $n$ values, i.e., up to 5000, the variations in the estimated $a$ values are well below 0.1.

### The relation between ${\boldsymbol{b}}\,$and the fracture growth rate ${\boldsymbol{a}}$ parameter

We consider the exponential magnitude distribution model, which results from the Gutenberg-Richter relation and reads:

5 $$f(M)=\beta {e}^{-\beta (M-{M}_{min})};F(M)=1-{e}^{-\beta (M-{M}_{min})}\,for\,M\ge {M}_{min},$$

$f(M)=F(M)=0$ for $M < {M}_{min}$, and $\beta =bln10$, where $M$ is the magnitude of events, $b$ is the Gutenberg-Richter constant and ${M}_{min}$ is the magnitude completeness. The model’s parameter is estimated using the maximum likelihood for discrete magnitude values^50,51^:

6 $$b=\frac{1}{ln(10)[\bar{M}-{M}_{min}]}$$

where $\bar{M}$ is the sample mean of the considered event magnitudes. The analysis is performed in the following steps:

1. Each FN is divided by the intervals of magnitude data with $a$ values: (i) $a\le 0.5$ and $a\, > 0.5$, (ii) $a\le 0.6$ and $a\, > 0.6$, and (iii) $a\le 0.4$ and $a\, > 0.7$.
2. For the selected groups of data, the exponentiality is tested, and the $b$ value is calculated.
3. Then, the difference in $b$ values for different FN growth periods associated with the same FN is tested.

We test the exponentiality of the magnitude distribution using the Anderson-Darling (AD) test^52^ (Table 3). Since the AD test is valid for continuous random variables, the magnitudes are randomized within their round-off interval of length 0.01 by the equation proposed by Lasocki and Papadimitriou^53^:

7 $${M}_{rand}={F}^{-1}\{u[F(M+\frac{\delta M}{2})-F(M-\frac{\delta M}{2})]+F(M-\frac{\delta M}{2})\}$$

where ${M}_{rand}$ are the randomized magnitudes $M$, $\delta M$ is the magnitude round-off interval, $u$, is a random value from the uniform distribution (0,1), $F(\cdot )$ is the cumulative distribution function (CDF) of magnitudes and ${F}^{-1}(\cdot )$ is the corresponding inverse CDF. The magnitude distribution does not follow the exponential distribution when the tested hypothesis of exponentiality is rejected at the significance level of 0.05.

**Table 3:** Results of the hypothesis for the exponentiality of the magnitude distribution testing using the Anderson-Darling test.

Fracture Network	Number of observations	*p* value	*b*	Number of observations	*p* value	*b*	*δb*
**TEST 1**	***a*** **≤ 0.5**			***a*** **> 0.5**			
1	35	0.366	1.184	43	0.801	1.171	0.01
2	27	0.389	1.072	41	0.152	1.132	−0.06
3	24	0.164	1.149	42	**0.017**	1.152	−0.00
4	23	0.126	1.039	50	0.076	1.048	−0.01
5	35	0.097	1.405	47	**0.012**	1.000	0.41
6	24	0.594	1.489	31	0.159	1.061	0.43
7	9	0.567	0.963	27	0.058	1.066	−0.10
8	32	0.287	0.817	18	0.191	1.019	−0.20
9	20	**0.001**	1.090	62	**0.045**	0.996	0.09
10	28	0.074	1.268	24	0.289	1.399	−0.13
11	13	0.203	1.701	67	0.232	1.270	0.43
12	32	0.828	1.114	52	0.104	0.948	0.17
13	—	—	—	28	0.161	1.528	—
**TEST 2**	***a*** ≤ **0**.6			***a*** > **0**.6			
1	35	0.365	1.184	43	0.801	1.171	0.01
2	41	0.115	1.065	27	0.420	1.214	−0.15
3	73	**0.017**	1.149	—	—	—	—
4	23	0.126	1.039	35	0.076	0.998	0.04
5	59	**0.021**	1.183	23	0.107	1.043	0.14
6	33	0.300	1.482	22	0.161	0.962	0.52
7	54	0.073	1.080	—	—	—	—
8	32	0.289	0.817	18	0.192	1.019	−0.20
9	32	**0.005**	1.103	50	0.071	0.975	0.13
10	35	0.103	1.335	17	0.227	1.324	0.01
11	13	0.207	1.701	67	0.232	1.270	0.43
12	40	0.986	1.163	52	0.103	0.948	0.22
13	—	—	—	28	0.160	1.528	—
**TEST 3**	***a*** ≤ **0**.4			***a*** ≤ **0**.7			
1	28	0.349	1.153	15	0.591	0.957	0.20
2	22	0.489	1.235	—	—	—	—
3	24	0.165	1.149	—	—	—	—
4	17	0.178	1.072	6	**0.006**	1.015	0.06
5	26	0.111	1.450	8	0.461	0.945	0.50
6	24	0.596	1.489	18	0.230	1.013	0.48
7	5	**0.044**	1.399	—	—	—	—
8	18	0.511	0.896	5	**0.017**	0.844	0.05
9	7	**0.0005**	3.162	31	0.178	0.863	2.30
10	7	0.228	1.309	9	0.181	2.537	−1.23
11	—	—	—	16	0.477	1.560	—
12	21	0.356	1.078	15	0.106	0.896	0.18
13	—	—	—	16	0.066	1.929	—

The columns are the fracture network code, number of data ($a\le {a}_{i}$), $p$ value from the AD test ($a\le {a}_{i}$)*, $b$ value ($a\le {a}_{i}$), number of data ($a > {a}_{i}$), $p$ value from AD test ($a > {a}_{i}$)*, $b$ value ($a > {a}_{i}$), and $\delta b$, i.e., difference: ${b}_{a\le {a}_{i}}-{b}_{a > {a}_{i}}$; *when $p < 0.05$, exponentiality is rejected at 0.05 significance.

We perform the *U* Mann-Whitney test to compare the $b$ values between the stages of the FNs with different growth rates. We compare only those pairs of $b$ values wherein the magnitude distributions for both stages are exponential. The results of the analysis are presented in Table 4. The test rejects similarities between the $b$ values of the stages of FNs with the $a$ parameter less than 0.4 and stages with the $a$ parameter greater than 0.7. The FN stages with a higher rate of growth are characterized by lower values of $b$, so they are more hazardous.

**Table 4:** The significance value of the *U* Mann-Whitney test comparing the $b$ values between the stages of the fracture networks with different growth rates as presented in Table 3.

	Sum of ranks for stages with ${\boldsymbol{a}}\le {{\boldsymbol{a}}}_{{\boldsymbol{i}}}$	Sum of ranks for stages with ${\boldsymbol{a}} > {{\boldsymbol{a}}}_{{\boldsymbol{i}}}$	Number of observations in stages with ${\boldsymbol{a}}\le {{\boldsymbol{a}}}_{{\boldsymbol{i}}}$	Number of observations in stages with ${\boldsymbol{a}} > {{\boldsymbol{a}}}_{{\boldsymbol{i}}}$	Test statistic *U*	*p* value	*p* adjusted for ties
TEST 1	140	113	11	11	47	0.393	0.401
TEST 2	122	88	10	10	33	0.212	0.218
TEST 3	88.5	47.5	8	8	11.5	**0.036**	**0.028**

Table contains the information about the test statistic, *U* statistic, and the asymptotic significance (2-tailed) *p* value.

### The influence of fracture network growth on the bulk modulus

The presence of cracks in rocks has a significant influence on elastic properties. The effective properties of a rock can be determined as a function of crack development parameters (e.g.,^54–56^). Generally, the bulk modulus for the cracked rock ($K$) is decreased compared to the uncracked rock modulus (${K}_{0}$). Generally, this relation has the form^38^:

8 $$\frac{K}{{K}_{0}}=\frac{1}{1+B\rho },$$

where $B$ is a constant and $\rho$ is the crack density. For the non-interactive regime, where each crack is unperturbed by other cracks, and the crack-generated strains are summed up, the Eq. (8) is linearized with respect to $\rho$, and stiffness is linear in crack density:

9 $$\frac{K}{{K}_{0}}=1-B\rho .$$

When $\rho \to 0$ two approximations (8) and (9) are compatible. For the non-interactive regime Walsh^57^ derived the relation between the bulk modulus $K$ and the bulk modulus of the uncracked material,$\,{K}_{0}$, for the isotropic case of randomly oriented penny-shapes cracks:

10 $$\frac{{K}_{0}}{K}=1+\frac{16(1-{\vartheta }_{0}^{2})}{9(1-2{\vartheta }_{0})}\rho ,$$

where ${\vartheta }_{0}$ is the Poisson ratio of the isotropic intact matrix. Using a standard value ${\vartheta }_{0}\,=\,0.25$, the above equation becomes $\frac{{K}_{0}}{K}=1+3.3\rho$.

The crack density depends on the crack interactions, crack orientations, its growth rate, the presence of the initial nuclei and the nucleation rate, the regime in which cracks grow, dilute (freely growing fractures) or dense (stopped fracture), or if they are fluid-filled or not (e.g.,^34,35,55,56^). The crack density can be approximated by the equation:

11 $$\rho =\frac{n(l){(\frac{\bar{l}}{2})}^{3}}{V},$$

where $n$ is the number of cracks in the volume $V$ and $\bar{l}$ is the mean crack length. An exponentially increasing growth rate of crack (Charles’ law) produces an exponentially decreasing distribution length (e.g.,^35^):

12 $$n(l) \sim {l}^{-a},$$

where $a$ is the growth exponent.

The non-interacting approach is generally considered as a good approximation when the crack density is low. For the high crack densities the interactions among cracks become significant and the non-interaction solutions may be inaccurate. To account for crack interactions, numerous improved approximate schemes have been proposed^57^. However, Kachanov^38^ reviewing the state of art of the effective elastic properties of cracked solids pointed out that in deriving the effective elastic modulus of cracked rocks, there are still issues needing to be resolved and the accuracy of approximate schemes should be examined since good accuracy derived for specific crack arrays may not apply to other arrays and loading conditions.

For simplicity here we assume the unit volume $V$, and ${(\frac{\bar{l}}{2})}^{3}$ is constant, then $\rho  \sim {\int }_{0}^{L}{l}^{-a}dl={L}^{1-a}\cdot \frac{1}{1-a}$. Thus $\rho  \sim A\cdot \frac{1}{1-a}$, where $A={L}^{1-a}\cdot {(\frac{\bar{l}}{2})}^{3}$ is constant. The $\frac{1}{1-a}$ function can be expanded to Maclaurin series as $1+a+{a}^{2}+\ldots$. In our case $a$ parameter is below 1 and this function can be approximated by $1+a$. Finally:

13 $$\frac{K}{{K}_{0}}=1-B\rho =1-AB(1+a)=1-AB-ABa.$$

We found that the most accurate results are when $\frac{K}{{K}_{0}}$ in (13) is approximated by $1-a$:

14 $$\frac{K}{{K}_{0}}=1-B\rho =1-AB-ABa\, \sim \,1-a.$$

When $a$ parameter approaches 1, the $A/V$ is close to 1. $B$ constant is derived empirically from the laboratory tests. In our case $B$ has to be from the interval $(0;0.5]$. The differences between the observed maximum magnitudes and upper limit derived from Galis *et al*.,^8^ and for the estimated effective bulk modulus, expressed by *RMSE* is 0.4 for $(1-a)$ coefficient, and 2.2 for the coefficients estimated from the Eqs. (8) and (9). The overestimations of maximum magnitude based on the Eqs. (8) and (9) can be the results of fracture compliance contribution. Here we consider fracture networks thus we expect the coplanar arrangements of fractures. For such fracture organization the dependence of fracture compliance can be stronger than mean radius cubed^38^. Based on these results we approximate the bulk modulus for the fractured rocks in The Geysers by a linear function of the fracture growth rate.

## Data Availability

The dataset is available on the IS-EPOS platform of the Core Service Anthropogenic Hazards developed in the framework of infrastructure projects IS-EPOS and the European Plate Observing System Programme (https://tcs.ah-epos.eu). All data needed to evaluate the conclusions in the paper are presented in this paper and in the Materials and methods. Additional data related to this paper may be requested from the authors.
