# Author Correction: Evidence for subcritical rupture of injection-induced earthquakes

**DOI:** 10.1038/s41598-020-63870-3

**Published:** 2020-04-17

**Authors:** Beata Orlecka-Sikora, Szymon Cielesta

**Affiliations:** Institute of Geophysics, Polish Academy of Sciences, Ks. Janusza 64 Str., 01-452 Warsaw, Poland

Correction to: *Scientific Reports* 10.1038/s41598-020-60928-0, published online 04 March 2020

This Article contains a typographical error in the Acknowledgements section.

“This work was supported under the Science for Clear Energy (S4CE) project”

should read:

“This work was supported under the Science for Clean Energy (S4CE) project”

